# Glucose-based biofuel cells and their applications in medical implants: A review

**DOI:** 10.1016/j.heliyon.2024.e33615

**Published:** 2024-06-25

**Authors:** Indrani Chakraborty, Richard T. Olsson, Richard L. Andersson, Annu Pandey

**Affiliations:** a Department of Chemistry, Chandigarh University, Mohali, 140413, India; b Department of Fibre and Polymer Technology, School of Chemical Science and Engineering, KTH – Royal Institute of Technology, Teknikringen 56-58, 100 44, Stockholm, Sweden

## Abstract

In glucose biofuel cells (G-BFCs), glucose oxidation at the anode and oxygen reduction at the cathode yield electrons, which generate electric energy that can power a wide range of electronic devices. Research associated with the development of G-BFCs has increased in popularity among researchers because of the eco-friendly nature of G-BFCs (as related to their construction) and their evolution from inexpensive bio-based materials. In addition, their excellent specificity towards glucose as an energy source, and other properties, such as small size and weight, make them attractive within various demanding applied environments. For example, G-BFCs have received much attention as implanted devices, especially for uses related to cardiac activities. Envisioned pacemakers and defibrillators powered by G-BFCs would not be required to have conventional lithium batteries exchanged every 5–10 years. However, future research is needed to develop G-BFCs demonstrating more stable power consistency and improved lifespan, as well as solving the challenges in converting laboratory-made implantable G-BFCs into implanted devices in the human body. The categorization of G-BFCs as a subcategory of different biofuel cells and their performance is reviewed in this article.

## 1 Introduction

Glucose is present in the tissues of all body organs and is constantly renewed by metabolism in biological fluids [1]. Glucose is produced in the body by hydrolysis of consumed food and then released into the bloodstream by active transportation through intestinal cells. It enters different metabolic pathways depending on the energy requirement of the cell. On entering the metabolic path, glucose produces energy through ATP utilizing Krebs's Cycle, Glycolysis, and oxidative phosphorylation [[2], [3], [4]]. Under the influence of these different physiological processes, glucose breaks down into carbon dioxide and water by oxidation. The conversion of glucose into carbon dioxide and water is synonymous with a very high energy density, which, according to theoretical calculations, is equivalent to approximately 16 kW per gram at the time of oxidation [5].

Biofuel cells (BFCs) can directly transform chemical energy into electrical energy through biochemical pathways by using enzymes or microorganisms as catalysts rather than expensive metal catalysts, making them economical alternatives to conventional fuel cells [6,7]. Using renewable power associated with glucose as the molecule also provides sustainability, and sources offer eco-friendliness [8,9]. Recently, it has been observed that using nanoparticles with conducting polymers can improve the performance of biofuel cells [10,11]. In a study by Saurabh Kumar et al., it was observed that when iron oxide nanoparticles are combined with poly (3,4-ethylene dioxythiophene): poly (styrene sulfonate) and used as the electrode, the electrochemical output and the stability of signal of the cancer biosensor were enhanced [12].

The biofuel cells can be categorized as microbial, enzymatic [13,14], and nanozymatic biofuel cells, reflecting the nature of energy conversion in the biofuel cell [15]. Glucose biofuel cells (G-BFCs) are a subclass of the enzymatic biofuel cell, which generates electrical energy by the oxidation of glucose at the anode and the reduction of oxygen at the cathode, yielding 24 electrons per glucose molecule. However, the number of electrons available for electricity harvesting may be substantially lower. A glucose biofuel cell's maximum power density, i.e., current and open-circuit voltage, determines its electrical output (watts per unit area), which accordingly best reflects the usefulness of biofuel cells [16]. The efficiency of glucose-based biofuel cells also depends on the type of circuit arrangements used in a pacemaker, the types of electrodes in the glucose biofuel cells, etc., and, by varying these parameters, the performance of glucose-based biofuel cells can strive towards improvement [[17], [18], [19]].

There are many applications of glucose-based biofuel cells. They can be seen running various devices like neurophysiological monitors, glucose-sensing contact lenses, cardiac pacemakers, drug delivery systems, and continuous glucose monitors [20]. The reason for using glucose is that it can act as a green alternative to conventional fuel sources. Besides, its abundant natural accessibility and constant production in blood or interstitial fluids make it a biocompatible, boundless, and independent power source inside any living body. Also, with the use of glucose, the size of the fuel cell is minimal, and the preparation of the fuel cells is naturally at a low cost [1,13]. Glucose is a biocompatible device-powering substrate since it is formed within bodies through various metabolic processes [21]. One of the most essential applications of glucose-based biofuel cells is within the field associated with cardiac support activities. The lifetime of the cardiac pacemakers powered by lithium batteries is limited to 10 years of the implant. Consequently, it must be replaced, resulting in infection risks from the expensive surgery.

**Scheme. 1:**
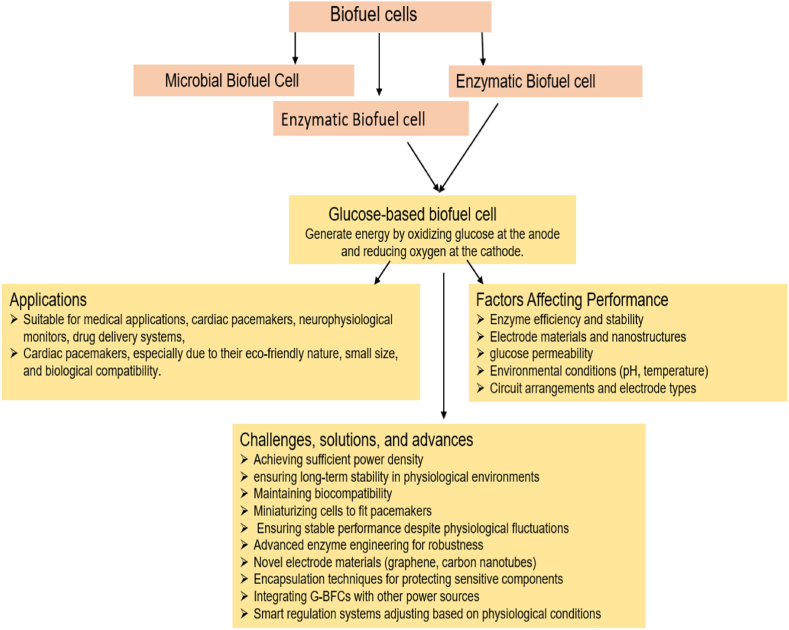
Overview of review.

Over the past decade, several reviews have addressed the fundamental principles and broad applications of biofuel cell-based bio-batteries, enzymatic biofuel cells, nanomaterial-based biofuel cells, and carbon-based biofuel cells. There remains a notable gap in the exploration of glucose-based biofuel and its medical implications, particularly in cardiac activities. This comprehensive review focuses on the various preparation methods and applications of glucose-based biofuel in medical devices, emphasizing its relevance to cardiac activities and medical devices related to heart health. An overview of work is represented in Scheme 1 [22,23]. We report the trends in developing G-BFCs and their applications in healthcare by reviewing the basic design of these devices and their pioneering applications in cardiac activities in the last ten years.

### 1.1 Biofuel cells (BFCs)

The idea of the development of biofuel cells first arose in the 1780s when Galvani observed a frog's leg convulsing on the application of electric current in his experiment [24]. In 1839, Grove et al. generated water and an electric current by reversing the process of electrolysis [25]. In 1910, Professor M.C. Potter discovered that the microorganism E. Coli could create electricity as a microbial fuel cell. In 1931, Cohen et al. explained that a larger voltage of more than 35 V could be obtained from microbial fuel cells if connected in electric series [26]. In the 1950s and 1960s, microbial biofuel cells were considered a technology that could be used in space flights to produce power from disposed waste. Cell-free enzyme arrangements were also utilized in biofuel cells in the late 1960s [27]. Consequently, over the years, further innovations and experiments were carried out to modify and develop biofuel cells, yielding better outputs regarding cell voltage and power. Overall, the performance of the biofuel cells is mainly affected by temperature, substrates, and the presence of catalysts, so depending on these variations, different biofuel cells have various applications [28].

Among different biofuel cells, microbial biofuel cells typically utilize microbes to convert wastes to energy; enzymatic biofuel cells use natural enzymes for energy production; enzymatic biofuel cells create energy with nanomaterials imitating natural enzymes and use waste as energy sources. Enzymatic and nonenzymatic biofuel cells show the most promising future in terms of usefulness in biomedical applications. The G-BFCs are the most important subclass of enzymatic and nanozymatic BFCs for employment in biosensors and implantable devices. Although the main focus herein is on the G-BFCs and their application in cardiac activities, it is vital to have a brief idea about the types and preparation of BFCs in general.

## 2 Types and preparation of biofuel cells

### 2.1 Microbial biofuel cells (MB/BFCs)

**Fig. 1:**
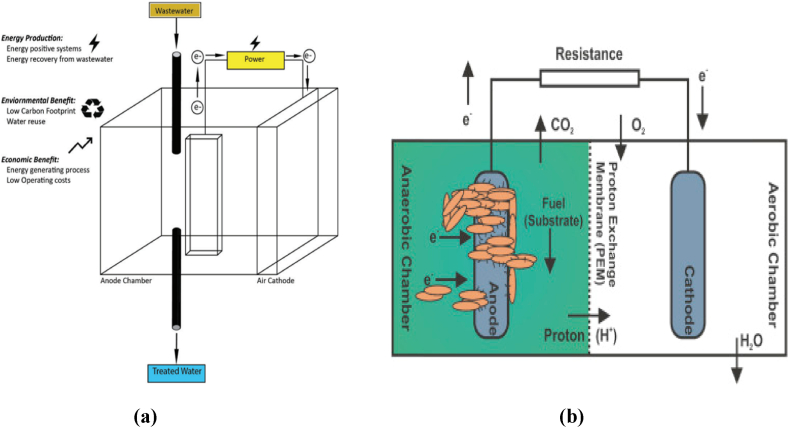
(a) A single-chambered microbial fuel cell for wastewater treatment system, with advantages of higher resistance to process disturbances [129, reprint with permission] (b) A double–chambered microbial fuel cell with cathode and anode chambers, allowing more efficient energy harvesting due to avoidance of reaction interference of electrons at opposing side electrode [19, reprint with permission].

In this type of biofuel cell, the oxidation of biological waste is catalyzed by utilizing a microorganism, and the biochemical energy is converted into electrical power [26]. On oxidation, the obtained electrons are transferred to the anode, producing a biofilm responsible for the activity at the anode. Depending on the nature of the transfer of electrons occurring in the MB/BFCs, they can be classified as mediator-based and mediator-less [29,30,31], reflecting the necessity of a mediator surface for the effective transfer of electrons. The MB/BFCs can also be divided into two categories based on their arrangement: single-chamber and dual-chamber MB/BFCs, see Fig. 1, providing advantages and disadvantages regarding their construction and efficiency [[26], [32],^33^].

These biofuel cells have many biofuel sources, such as sewage water, soil formed as marine sediment, dirt associated with freshwater, and activated slush, demonstrating their usefulness in environmental technology [34,35]. The maximum power output exhibited by an MB/BFC to date is 5.61 μW/cm^2^ [26]. MB/BFCs have risen to such prominence that they stand a chance as an eco-friendly and economical alternative to conventional energy production methods, using their biocatalytic abilities with suitable microorganisms [36].

Besides the energy consideration with the MB/BFCs (above), Zhao et al. demonstrated sediment-based MB/BFCs for simultaneously detecting chromium (VI) ions in industrial wastewater. The chromium (VI) ions were reduced at the cathode, and MB/BFCs effectively monitored the chromium ion content between 0.2 g/L and 0.7 g/L [37]. Furthermore, Prévoteau et al. prepared an MB/BFC with microbial cathodes that reduce oxygen. It was utilized to detect toxic contents like Hg (II), Cr (VI), and Pb (II) in tap water [38]. In another study, Schneider et al. found a quick method for analyzing β-lactam antibiotics by accommodating an MB/BFC within the panel system while generating electrons. Owing to the MB/BFC integration, the system could assess the effectiveness of the antibiotics within two to 4 h.

In contrast, the traditional measurement method takes one to two days to evaluate effectiveness [39]. One of the research groups, a cathode-shared MB/BFC sensor array, was prepared for sensing acidic toxicity [40]. Recently, Li et al. designed a plant-based MB/BFC to measure the damage caused by acid rain. This MB/BFC was based on the concept that rhizosphere microorganisms produced electrochemical signals after a minute of acid rain occurrence and was utilized to minimize the damage caused by acid rain [41]. In summary, using energy-producing MB/BFCs frequently comes with additional benefits related to environmental sensor functionality.

### 2.2 Preparation of microbial biofuel cells

The single-chambered MB/BFCs are primarily made up of an anode and an air cathode, see Fig. 1a. However, the basic setup used for explaining the working mechanism of most of the MB/BFCs is the dual-chamber design, with an anodic chamber and a cathodic chamber, see Fig. 1b. The anode and cathode are made up of glass, polycarbonate, or polymethyl-methacrylate and have an electrode made up of carbon paper, carbon-cloth, graphite, graphite felt, platinum, platinum black, or reticulated vitreous carbon respectively. These two chambers are partitioned and employ a proton exchange membrane called PEM [42]. The membrane is one of the essential parts of the dual chamber because it prevents mediators and substrates from diffusing to other electrodes, interfering with the reaction, and minimizing undesired flux between electrodes while retaining their ionic and chemical conjugation [43]. The PEM is usually made up of Nafion or ultrex polymeric materials, and lowering undesired flux is of utmost importance to favor efficient electron harvesting [44]. The anodic chamber comprises organic materials that microorganisms degrade to produce energy and electrons. For the completion of the circuit, the cathodic section is made up of an electron acceptor of high potential. The power density can be increased by utilizing a stable electron acceptor such as oxygen, nitrogen-containing compounds, metal-containing ions, azo dyes, nitrogenous aromatic compounds, and chlorophenols that do not interfere with or have toxic effects on microorganisms or other system components. Oxygen is preferred as an oxidizing agent and electron acceptor at the cathode because of its abundance and non-toxic nature. Ferricyanide may be used as an alternative to the oxygen [[44], [45], [46]].

### 2.3 Enzymatic biofuel cells (ENZ/BFCs)

In this type of biofuel cell, the oxidation of the substrate is catalyzed by employing a specific natural enzyme, and subsequently, the biochemical energy obtained is converted into electrical energy. Enzymatic biofuel cells have quite an extensive range of biofuel sources such as sugar derivatives, viz. fructose, glucose, sucrose, and alcohols like ethanol and methanol. In addition, different organic acids and salts, such as sulfites, can also be used as biofuel sources. Unlike the MB/BFCs, these ENZ/BFCs have advantages. They do not require a membrane owing to the high specificity characteristics of natural enzymes towards the biofuel substrate. Among all other ENZ/BFCs, G-BFCs are the most popular and widely investigated [[47], [48], [49], [50]]. EBFCs are highly effective in pacemakers and glucometers due to their high activity under mild conditions, offering new possibilities for portable, cost-effective, and online diagnostic equipment. The discovery of EBFC-driven artificial muscles marks a new era in EBFC research, highlighting their potential for developing highly biocompatible artificial organs.

### 2.4 Preparation of enzymatic biofuel cells

**Fig. 2:**
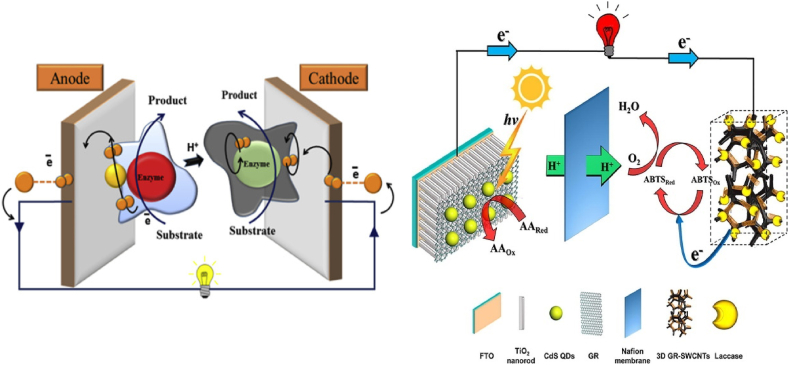
Schematic diagram of (a) Demonstration of an Enzymatic Biofuel Cell retaining a mediator-driven anode and a direct electron transfer-based biocathode. [121, reprint with permission] (b) Representation of the principle of Nanozymatic Biofuel cells prepared with nanomaterial GR–CdS QDs/TiO2 NRAs photoanode and 3D GR–SWCNTs–laccase hybrid bio-cathode [127, reprint with permission].

In an ENZ/BFC, the setup is made up of an anode, cathode, and counter electrode, without any membrane, see Fig. 2a. The immobilized enzyme like GO_x_, GDH_y,_ etc. oxidizes the biofuel, and the electrons generated in the oxidation are passed to the anode, from where the electrons further get transferred to the cathode under the application of potential at the anode [14]. By utilizing these electrons, the enzyme at the cathode converts oxygen to water, and subsequently, these chemical reactions produce bioelectricity used to power the ENZ/BFCs. The electron transfer through the electrodes occurs directly or indirectly. The direct mode of electron transfer is known as DET (direct electron transfer), and the indirect mode of transfer of electrons is known as MET (mediated electron transfer). In the DET mechanism, the transfer of electrons between the surface of the electrode and the enzyme takes place without a mediator. In contrast, in the MET mechanism, the mediators (methylene blue, pyrroloquinoline quinone (PQQ), ferrocene-methanol, ferrocene-monocarboxylic acid, and ferrocene-carboxaldehyde) are incapacitated on the surface of electrodes. Electron transfer occurs between the surface of the electrode and the enzyme. Accordingly, the power density (cell performance) will depend on the mode of electron transfer [5,51]. Conclusively, MET generates a greater current density (high power density) but needs the immobilization of extra components, which makes the process much more unstable and complicated. That is why DET is expected to show more excellent stability in current production with less barrier in its structure [[24], [27], [52], [53]].

### 2.5 Nanozymatic biofuel cells

Nanotechnology enables the creation of nanoscale electronic devices for high-performance biofuel cells. Nanoenzymatic biofuel cells, using enzyme-mimicking nanomaterials, can continuously produce electricity, making them ideal for powering implantable medical devices. This technology offers promising applications in biomedical devices, environmental monitoring, and portable electronics. Nanozymes are nanomaterials (Fig. 2b) that mimic natural enzymes' activities. These electrode materials include noble metals, carbon, bi- or tri-metallic alloys, hybrids like metal-organic frameworks, and metal oxides [50,54]. Like ENZ/BFCs, these nanozyme-based biofuel cells (NBFCs) are membrane-less. Among different types of NBFCs, glucose-based NBFCs have carved out a significant area for themselves in the research prospect [[55], [56], [57]].

### 2.6 Preparation of nanozymatic biofuel cells

In nanozymatic biofuel cells, the electrons are produced at the nanomaterial-based anode and flow through an electrical circuit to the cathode made of another nanomaterial. The oxidation of the biofuel, which is the substrate, is catalyzed by using an enzyme mimicking a particular natural enzyme, resulting in the chemical energy being converted into electrical energy. The most common methods for synthesizing nanozymes are top-down and bottom-up physicochemical material synthesis techniques. In the top-down process, initiator compounds are scaled down for preparation through ball milling, nanolithography, sputtering, and thermal decomposition. At the same time, the bottom-up approach is a wet-chemical technique that includes the sol-gel method, reverse micelle, chemical vapor, pyrolysis, biosynthesis, and microwave-supported. The bottom-up approach is more efficient as it is applied to prepare various nanozymes [50].

### 2.7 Application of biofuel cells

The most critical applications of biofuel cells are in managing effluents, bioelectronics, medical devices, etc.

MB/BFCs are essential for managing effluents from various sources, such as localities and pharmaceutical and textile industries. Wastewater serves as the substrate for producing electricity through the action of microorganisms. MB/BFCs have also been employed to produce electric power by replacing the chemical energy inside the chemical molecules with biomass and enhancing the process via microorganisms [28]. Also, secondary fuels like hydrogen can be generated by altering MB/BFCs a bit [58].

BFCs show their application in different kinds of bioelectronic devices. A study by Bandodkar et al. demonstrated that an LED lamp could be powered by deriving energy from ENZ/BFCs from sweat. In this ENZ/BFC, the lactate oxidase immobilized anode interacted with the human effort to generate electrons, and silver oxide fabricated Carbon nanodots (CNATs) facilitated smooth electron passage, thereby producing a significant voltage of 0.5 V and a power density of nearly 120,000 μWcm^−2^ [59]. ENZ/BFCs have also been used in portable devices by modifying them into several archetype transformations such as microfluidic prototypes [[60], [61], [62], [63], [64]], biofuel cells based on the paper [[65], [66], [67]], biobatteries [[68], [69], [70], [71]], etc.

In addition to the applications mentioned above, another significant application of BFCs is power generation in implantable medical devices such as cardiac pacemakers, glucometers, contact lenses, etc. BFCs in implantable medical devices may become essential due to their non-toxic, renewable, and environment-friendly properties. Moreover, if such an alternative power source can be employed in the biomedical field, it will open new avenues of better living standards, avoiding repeated surgery activity to replace batteries. Recently, metal electrodes were used after being modified into nanoparticle-based electrodes for the nanozymes, generating more stable power output than conventional ones [[72], [73], [74], [75], [76]]. The NANO/BFCs are envisioned to substitute the ENZ/BFCs in implantable medical devices. In addition to the applications above, the hydrogen byproducts from BFCs have been thought to be utilized in the transportation sector and for producing electricity in fuel cells [77]. Hydrogen energy has also been considered for application in robotics to generate power using artificial symbiosis [78].

### 2.8 Glucose-based biofuel cell

**Fig. 3:**
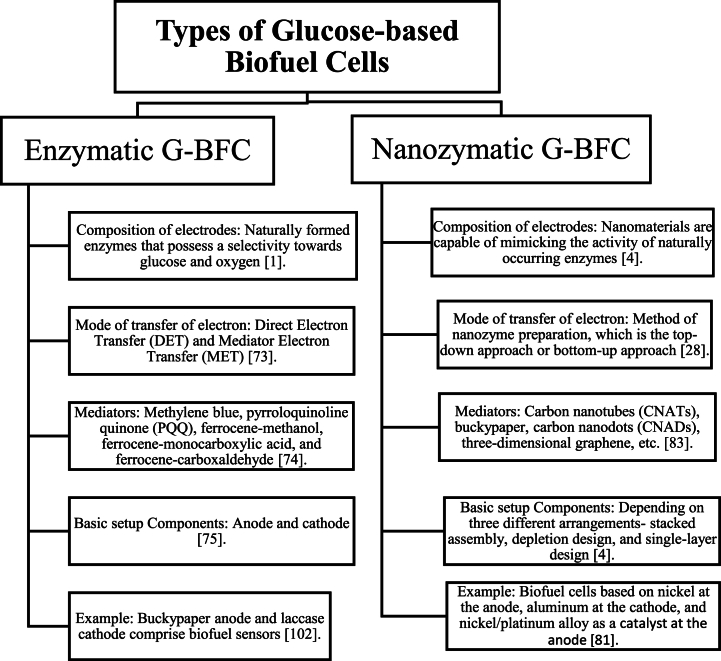
Classification of G-BFCs based on the nature of anode.

Among all types of BFCs, G-BFCs have carved out a distinct place for themselves, especially in implantable applications, and can be categorized under the enzymatic or nanozymatic BFCs, see Fig. 3. These BFCs consist of bioelectrodes where glucose, the substrate, undergoes the process of oxidation at the anode by the catalytic activity of an enzyme and subsequently produces electrons, which are responsible for creating electricity. Several factors affect the cell output efficiency, namely-the concentration of fuel, the mode or mechanism of transfer of electrons, the action of the enzyme, the potential of the cell, and others [21,79,80].

The reactions involved in a G-BFC are [13]:

Complete gluc ose oxidation: C_6_H_12_O_6_ + 6O_2_ → 6CO_2_ + 6H_2_O

Anodic glucose oxidation (GOx): C_6_H_12_O_6_ → C_6_H_10_O_6_ + 2H^+^ + 2e^-^

$$\text{Cathodic}\;\text{oxygen}\;\text{reduction}\;\left(\text{laccase}\right):\frac{1}{2}\;O_{2}+2H^{+}+2e^{-}\;\to \;H_{2}O$$

$$\text{Overall}\;\text{GFC}\;\text{reaction}:C_{6}H_{12}O_{6}+\frac{1}{2}\;O_{2}\;\to \;C_{6}H_{10}O_{6}+H_{2}O$$

### 2.9 Types and preparation of glucose-based biofuel cells

Depending on the type of anode utilized in the G-BFCs, the G-BFCs can be categorized under two broad headings-1) Enzymatic Glucose Biofuel Cells (ENZ/G-BFCs) and 2) Nanozymatic Glucose Biofuel Cells (NANOENZ/G-BFCs). Though NANOENZ/G-BFCs seem to be a subclass of ENZ/G-BFCs, they should be given adequate importance separately because NANOENZ/G-BFCs are made up of nanozymes, which are nanomaterials possessing the ability to imitate natural enzyme's activities. Besides, NANO/G-BFCSs exhibit higher cell voltage and power density than ENZ/G-BFCs.

### 2.10 Enzymatic Glucose Biofuel Cells (ENZ/G-BFC)

Enzymatic Glucose Biofuel Cells (ENZ/G-BFCs) use electrodes made up of naturally formed enzymes that possess a selectivity towards glucose and oxygen for the oxidation of glucose and reduction of oxygen [1]. The electrode constituents’ specific parameters, such as suitable biocatalyst capable of carrying out physical and chemical transformation to produce energy from glucose, enough operative surface area so that the biocatalyst experiences a more significant number of spots for adsorption, robustness of the material for longevity, easy fabrication, etc. [[81], [82], [83], [84], [85]]. Some of the mediators used for the oxidation of glucose oxidase (GOx) are methylene blue, which can generate a power density of approximately 1500 mW/m2; polydopamine (PDA), which has as excellent biostability and is also harmless to the human body, pyrroloquinoline quinone (PQQ) and ferrocene derivatives are popular because of their capability to donate electrons to acceptors other than dioxygen [[24], [27], [52], [53]].

In one of the studies by Leech and group., integrating multiple enzymes at the anode yielded twenty-four electrons per molecule of glucose and generated a cell output of 1.24 V at 298 K, whereas employing a single enzyme at the anode produced two electrons per molecule of glucose and lead to the production of cell output of 1.18 V. Hence, it concludes that the number of enzymes assembled at the anode of E/GBFCs affected the cell output voltage. Multiple enzyme assembly showed better output performance than single enzyme one [86].

### 2.11 Preparation of Enzymatic Glucose Biofuel Cells

The basic setup of an ENZ/G-BFCs comprises an anode made up of an enzyme that can carry out the process of oxidation, such as GOx, glucose dehydrogenase (GDHy) or glucose dehydrogenase reliant on pyrroloquinoline quinone (PQQ/GDHy) and a cathode of multicopper enzymes to carry out the process of reduction, e.g. laccase, bilirubin oxidase, or of same material. Davis and Yarborough were the first to utilize GOx at the anode of an ENZ/G-BFC in 1962 [[87], [88], [89], [90], [91],^92^]. The more conventional setup without membrane was first introduced by Katz et al. comprising an anode utilizing surface reconstructed GOx single layer and a cathode operating surface rebuilt Cytochrome C/Cytochrome oxidase pair. Glucose was oxidized to gluconic acid at the anode, and oxygen reduction to water was reduced at the cathode. At 40 mV and pH 7, this G-BFC exhibited a power density of 4 μWcm^−2^ [93]. Another setup was explained by Tsujimura et al. where GDHy was employed as an anode in an ENZY/G-BFC without any compartment. At pH 7, a power density of 58 μWcm^−2^ was exhibited by this setup [1].

Further advancement in preparing ENZ/G-BFC for improved efficiency led to modifications of the electrodes, e.g., the natural enzymes were combined with nanomaterials, polymers, or other materials like metal-based hydrogels. One of the more significant setups contributing to the advanced preparation of E/GBFC was proposed by Heller, in which a redox hydrogel made of osmium metal was used for immobilizing GOx at the anode, and copper oxidase at the cathode produced a large voltage (open circuit) of 0.8 V. In a study by Ivnitski et al. PQQ/GDHy was combined with a single-walled carbon nanotube at the anode to increase power density output [94]. Rouillard and researchers investigated the option to harvest energy from enzymatic biofuel cells for an extended period. Heller has reported a mixed operational/storage stability of an MWCNT-based glucose biofuel cell (G-BFC) over one year [95].

Besides selecting the electrodes in the preparation of ENZ/G-BFCs, the type of circuit arrangement also plays an important role. In a two-dimensional, several-level, microfluidic ENZ/G-BFC presented by Renaud et al. it was demonstrated that when a series configuration was used, the highest power it showed was 12.62 μWcm^−2^. Still, when the parallel arrangement was used, the highest power offered was 13.37 μWcm^−2^. Furthermore, Szczupak et al. showed an ENZ/G-BFC with (PQQ/GDHy) combined buckypaper anode and laccase cathode, which in series configuration exhibited circuit voltage of 0.8 V, short circuit current of 25 μA, and highest power of 5.2 μWcm^−2^. In contrast, the parallel design showed a circuit voltage of 0.36 V, a short circuit current of 300 μA, and the highest power of 37 μWcm^−2^ [[96], [97], [98]].

### 2.12 Nanozymatic Glucose Biofuel Cells (NANO/G-BFCS)

Nanozymes can be defined as nanomaterials capable of mimicking the activity of naturally occurring enzymes, and consequently, NANOENZ/G-BFC relies on their nature. Several nanozymes, such as metal chalcogenides, nanocarbon, nanocarbon composites, and others, have been known, each with its different catalytic activity [16]. The nature of the catalytic activity and enzyme-mimicking ability is related to the method of nanozyme preparation, which is a top-down or bottom-up approach, the latter being the more efficient one [50].

NANO/G-BFCS refers to the G-BFCs comprising electrodes of nanozymes—the ability to imitate the catalytic function of GOx and catalase enzymes at the anode. The main phenomena occur at the anode where oxidation of glucose, without forming hydrogen peroxide, appears along with laccase enzyme imitating enzymes at the cathode. Various enzymes observed imitating GOx, laccase, or catalase enzymes can be classified into two categories depending on their composition: metal oxide enzymes and noble metal enzymes. The performance efficiency of this kind of G-BFC depends on the selectivity of the catalysts at the anode, nanozyme material employed at the anode, or both electrodes and an electrode's definite area of the surface [50].

Apart from the conventional enzymes like glucose oxidases (GOxs) and glucose dehydrogenases (GDH), bilirubin oxidase (BOD) and laccase, and oxidoreductase enzyme components, some new features have recently been used in NANO/G-BFCS, which are carbon-based nanomaterials-carbon nanotubes (CNATs), bucky paper, carbon nanodots (CNADs), two-dimensional graphene. Buckypaper can be described as a film-like component with features like thinness, featherweight, and a flexible and compact structure, enabling it to be molded into any desired form. The CADs particles are a special kind of carbon-based nanomaterial that are distinct and possess an apparent spherical shape [[99], [100], [101], [102]].

It has been observed from the study of Huang et al. that if nanozymes that are smaller, about 10 nm, are used in NANO/G-BFCS, they usually exhibit more significant catalytic action than nanozymes that are larger [103]. Also, from the study of Yang and the group, if NANO/G-BFCS utilizes a nanozyme with a more significant number of activated crystallographic surfaces, it will experience more significant catalytic action for a nanozyme possessing a lesser number of the activated crystallographic textures. In NANO/G-BFCS, a catalase-mimicking nanozyme alone, if used, cannot serve the purpose of its employment. Still, the aim will be served if an enzymatic nanozyme showing GOx and catalase-mimicking properties is used [[104], [105], [106]].

### 2.13 Preparation of Nanozymatic Glucose Biofuel Cells

**Fig. 4:**
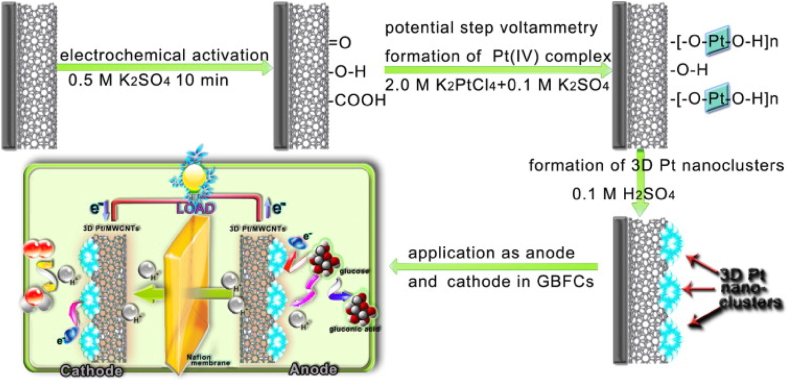
Represents the three-step process of preparing flowerlike 3D Pt/MWCNT catalysts with a detailed view of GBFCs-based 3D Pt/MWCNT electrodes [119, reprinted with permission].

The preparation of NANO/G-BFCS can be done mainly depending on three different arrangements-stacked assembly, depletion design, and single-layer design [[103], [107], [108], [109], [110]]. In the stacked assembly setup, diffusion of the compounds reacting to form products occurs in the G-BFCs from both sides. Ahead of the cathode, a hydrophobic membrane is positioned to stop the glucose from getting through and subsequently interfere with oxygen reduction. At the anode, a porous, thick noble metal is employed [16]. In the depletion design arrangement, the cathode comprises a catalyst, for example, activated carbon, which is unreactive towards glucose and will only selectively take up oxygen and thus reduce it. The cathode is placed ahead of the anode to generate an environment free of oxygen there [[111], [112]]. In the single-layer design, the anode and the cathode are positioned alongside. So, there is no need for the layers of electrodes to have a sideways outline, for which enough lenience of anode towards oxygen's existence in body fluids is required. An example of an anode used in such a setup is an anode made up of Raney platinum film [109]. In one of the other studies by Zhao et al. a new morphology was investigated by 3D print technology (see Fig. 4). In their work 3D flowerlike platinum (Pt) nanoparticle clusters were electrodeposited on the surface of multiwalled carbon nanotubes (MWCNTs) with better electrocatalytic activity and better stability for glucose oxidation reaction (GOR) and oxygen reduction reaction (ORR) [113].

Among all the demonstrated designs, the single-layer design is considered the most convenient and more advantageous than other designs [16]. This is because of its easy construction, decreased width, and simplified application in implantable devices. An example of the single-layer design is a G-BFC prepared by Oncescu et al. where they employed nickel at the anode, aluminum at the cathode, and nickel/platinum alloy as a catalyst at the anode [114]. This alloy showed more selectivity towards glucose than other enzyme-based catalysts. This setup was economical as it can be applied to implantable devices as coating layers [115].

### 2.14 Application of glucose-based biofuel cells

One of the most important categories of biofuel cells is G-BFCs because of their extensive potential applications [116,117]. Some of the most significant are biosensors, implanted devices, medical devices, and more (Table 1).

**Table 1:** Application of glucose-based biofuel cells.

Type of G-BFC	Sensing material	Application	Ref.
Enzymatic G-BFC	GOx modified with polyvinyl imidazole-[Os(bipyridine) 2Cl] composites and CNATs & Bilirubin oxidase modified with carbon fabrications	Self-powering biosensor for observing glucose levels in the blood	[118]
Nanozymatic G-BFC	Buckypaper and modified with a hydrogel matrix & Bilirubin oxidase and 1-pyrene methyl anthracene-2-carboxylate	Self-powering contact lenses	[119]
Multilevel nanozymatic G-BFC	Thin films of polyester	As generators in micro-sized or nano-sized bioelectronic devices.	[96]
Enzymatic G-BFC	Carbon fiber microelectrodes collaborated with a neutral red mediator and GOx	Utilized in catheters	[120]
Enzymatic G-BFC	GOx-3D carbon nanotube matrix modified anode and laccase-3D carbon nanotube matrix modified cathode with naphthoquinone mediator	Powering LED lamp	[121]
Hybrid enzymatic G-BFC	GOx, cobalt phthalocyanine, 1-pyrene-butyric acid modified with buckypaper anode and MnO_2_, PyBA modified with buckypaper cathode.	Biosensors in the food industry	[122]
Enzymatic G-BFC	Screen printed modified with bilirubin oxidase and GDHy	Powering electrolyzers	[123]
Enzymatic G-BFC	(PQQ/GDHy) based buckypaper modified anode and laccase based buckypaper modified cathode	Powering cardiac pacemakers	[124]
Enzymatic G-BFC	GDHy-methylene green combined and single-walled carbon nanotube modified anode and laccase-single-walled carbon nanotube cathode.	Neurochemical sensing	[125]

In a biosensor study by Yoshino et al. a G-BFC employing GOx at the anode modified with polyvinyl imidazole—[Os(bipyridine)2Cl] composites and CNATs and bilirubin oxidase cathode modified with carbon fabrications showed the potential to serve as a self-powered biosensor for observing glucose levels in blood [118,126,127].

Another important application of G-BFCs is the development of self-powering contact lenses. In a research study, it was observed that a G-BFC utilizing an anode made of buckypaper and modified with a hydrogel matrix and a cathode prepared with bilirubin oxidase and 1-pyrene methyl anthracene-2-carboxylate generated a power density of 8.01 μWcm^−2^ at a circuit voltage of 0.413 V at 35 °C in man-made tear solution [[119], [128], [129]].

The G-BFCs have also been implemented as generators in micro-sized or nano-sized bioelectronic devices. For example, Desmaële et al. prepared a multilevel G-BFC without a membrane, employing thin polyester films at the electrodes, which provided enough power to wirelessly transmit temperature readings to a remote computer [96]. Besides being used in bioelectronic devices, the G-BFCs are also used as implantable devices, e.g., in cardiac pacemakers [130,131]. In a study undertaken by Scott et al. an enzymatic G-BFC made up of a PQQ/GDHy-based buckypaper anode and a laccase-based buckypaper cathode displayed an open circuit voltage of 0.47 V and current density of 0.83 mAcm^−2^, adequate enough to power cardiac pacemakers [124].

Another application of G-BFC is in monitoring neurophysical actions, neurochemical sensing, etc. Cheng et al. prepared an enzymatic G-BFC employing GDHy in combination with methylene green. They modified it with a single-walled carbon nanotube as the anode and laccase-single-walled carbon nanotubes as the cathode. It generated an open circuit voltage of 0.78 V and a peak power density of 48μWcm^−2^ at 0.40 V [125]. Besides, these biofuel cells can also be utilized in medical devices. For instance, the G-BFC prepared by Sales et al. can be employed in catheters. The G-BFC was made of carbon fiber microelectrodes in collaboration with the neutral red mediator and GOx [[120], [132], [133], [134]].

The G-BFCs have also been used to power electrical devices like LEDs (light emitting diodes), digital thermometers, etc. For example, in a study by Zebda et al. it was seen an ENZ/BFC, specifically a G-BFC consisting of GOx-3D carbon nanotube matrix modified anode and laccase-3D carbon nanotube matrix modified cathode, with naphthoquinone produced a cell output of 0.57 V and a power output of 193.5 μWcm^−2^ which could drive a LED [121]. Apart from these, the G-BFCs have been utilized to produce glucose biosensors, which are subsequently used in food industries and more [122]. An instance of such utilization can be stated from the study of Hao et al. where a mediator-less G-BFC possessing GOx, cobalt phthalocyanine, 1-pyrene-butyric acid (PyBA) modified with bucky paper anode, and manganese dioxide (MnO2), PyBA modified with bucky paper cathode.

G-BFCs have other applications in powering electrolyzers. In a study performed by Pinyou et al. a G-BFC employing screen-printed anodes and cathodes with modification of bilirubin oxidase and GDHy generating circuit voltage of 0.567 V and power density 6.8 μWcm^−2^ powered an electrolyzer [123].

### 2.15 Application of glucose-based biofuel cells in cardiac activities

Glucose-based biofuel cells can power implantable cardiac devices like pacemakers and defibrillators, ensuring continuous operation without frequent battery replacements. This is crucial for maintaining heart rhythm stability and preventing life-threatening arrhythmias. This real-time data can aid in the early detection of cardiac abnormalities or ischemic events, improving patient outcomes. In emergencies like cardiac arrests, glucose-based biofuel cells can power implantable or wearable devices that deliver immediate therapeutic interventions, such as electrical stimulation or drug delivery, to restore normal heart function before medical help arrives. Glucose-based biofuel cells can support long-term implantable sensors for patients with chronic heart conditions that monitor cardiac health indicators like blood glucose levels and lactate concentrations. This data can facilitate personalized treatment plans and disease management strategies. By placing particular emphasis on applying glucose-based biofuel cells in cardiac activities, researchers and healthcare professionals can unlock new possibilities for advanced cardiac monitoring, emergency response, and personalized therapeutic interventions, ultimately improving the quality of care for patients with heart-related conditions.

One of the most significant applications of G-BFCs lies in the domain of cardiac devices, including cardiac pacemakers (CPs) and cardiac defibrillators (CDs) [135,136]. CPs treat irregular heart rhythms, while CDs refer to devices mainly used for identifying and treating arrhythmias to prevent death. Currently, CPs are powered by lithium batteries, which have drawbacks such as a limited lifespan of five to ten years, necessitating replacement after that period. This replacement process is costly and has a significant infection risk [20,137,138].

The G-BFCs offer a sustainable alternative for powering devices like CPs and CDs, utilizing electric power obtained from glucose oxidation. The feasibility of incorporating G-BFCs into CPs or CDs depends on several factors, including the circuit arrangement, the size of the G-BFC, the amount of power produced, and the voltage generated. Typically, a CP requires about 5–10 μWcm^−2^ of power, while a CD demands slightly less [1,139].

Several G-BFCs have demonstrated the potential of G-BFCs as an eco-friendly power source for CPs and CDs. In a study by Katz et al. a cardiac pacemaker circuit utilized a G-BFC and a charge pump DC-DC boost-converter, producing a stable 3 V output equal to that of a lithium-ion battery. In another experiment, a single G-BFC implanted within a lobster displayed open-circuit voltage and current density of 0.47 V and 0.83 mAcm^−2^, respectively, enough to provide power and make a cardiac pacemaker work [24]. Despite its short-lived power supply, this G-BFC proved biocompatible, as its implantation in the lobster caused no surgical complications [140].

Furthermore, Zhao et al. demonstrated a nanozymatic G-BFC employing 3D flower-like platinum nanoparticles deposited on multi-walled carbon nanotubes as electrodes on a membrane of Nafion. This configuration exhibited stability in performance for about 30 continuous days and promising biocompatibility due to its small size while providing a power density and open-circuit voltage of 2.3 μWcm^−2^ and 0.70 V, respectively. Conclusions could, however, not be assessed regarding its biocompatibility and possible surgical complexity as it was evaluated in vitro [113].

In one of the studies, Kloke et al. described nanozymatic G-BFCs with porous platinum electrodes and cyclic platinum and copper alloy deposition to fabricate G-BFCs exhibiting a power density of 5.1 μWcm^−2^. Kolke's G-BFC showed stability in power performance for around 90 days, with a 0.8 % decay rate every day. Although it showed promising biocompatibility, owing to copper deposition as residue, which can further cause physiological disorders [141].

Weidlich et al. demonstrated a double-chambered enzymatic G-BFC implanted in a dog, having purified aluminum in the form of the anode and activated carbon in the form of a cathode, generated a power density of 4 μWcm^−2^at an open voltage of 0.575 V for 150 days, validating that reliable power can be obtained for long times [16].

Recent research has explored the implantation of glucose-based fuel cells in rats, utilizing interstitial fluids rather than blood for power. The cells consisted of structured amalgamated graphite discs, comprising GOx and ubiquinone as anode and polyphenol oxidase (PPO) and quinone as a cathode, in an electrode system that yielded a steady power of greater than 7.52 mW/mL and peak power of 24.4 mW/mL. Stability in the output was observed for around 11 days, along with promising biocompatibility and no surgical complexities. Further, when a 4 mL dialysis tube modified with expanded polytetrafluoroethylene was inserted in the retroperitoneal interstellar together with the G-BFC, the life span of the G-BFC was extended up to 3 months with good biocompatibility [142]. Mano et al. reported a comparable application on the power production of bio-fuel cells in which bioelectrocatalyst-coated carbon-fiber electrodes were the primary fuel cell electrode component [143]. The electrocatalysts of this study were “wired” enzymes (polyanions) consisting of GOx and BOD with redox potentials of −0.19 and + 0.36 V, respectively. The power output of 1.9 μW for a week was considered sufficient for powering low-power CMOS circuits, with an operating voltage of 0.52 V being adequate for low-voltage CMOS/SIMOX integrated circuits. Additionally, when implanted in a grape, a living organism, the cell retained 78 % of its initial power output of 1.1 μWcm^−2^.

In another recent study of glucose-based enzymatic fuel cells, Yazidi et al. reported a maximum power of 44 μWcm^−2^for their glucose biobattery, which was achieved as a significant improvement over conventional enzymatic fuel using BOD by the use of a solid-state Prussian Blue (PB) thin-film cathode coated onto multiwall carbon nanotubes on carbon paper in the biofuel cell [144]. The consistency in power generation of the glucose-based biobattery could be seen till 20 cycles of charging-discharging [144].

The present performance characteristic described by researchers about BFC is directed towards use in pacemakers, thus primarily related to allowing the G-BFC to operate or mimic accurate operation with steady power output for more extended times, as described above, currently ranging from 5 h to 190 days [[124], [145], [146]]. Table 2 summarizes the performance of G-BFCs in terms of sensitivity, stability, and biocompatibility, which clearly indicates the advantages and disadvantages of G-BFCs in cardiac activities.

**Table 2:** Performance of glucose-based biofuel cells in cardiac activities.

Type of G-BFC	Open Circuit Voltage	Current Density/Power Density	Stability	Sensitivity	Biocompatibility	Ref.
Single G-BFC	0.47 V	0.83 mA/cm^2^	Short-lived power supply	–	Promising biocompatibility	[24,140]
Nanozymatic G-BFC	0.70 V	2.3 μW/cm^2^	30 days	pH: 5–7.5 Glucose: 0–1 M	Inconclusive	[113]
Nanozymatic G-BFC	–	5.1 μW/cm^2^	90 days with 0.8 % decay per day	pH: 7.4 Glucose: 3 mmol L^−1^	Inconclusive	[141]
Double-chambered enzymatic G-BFC	0.575 V	4 μW/cm^2^	150 days	–	Good biocompatibility	[16]
Enzymatic G-BFC	–	7.52 mW/mL −24.4 mW/mL	Initially 11 days; with polytetrafluoroethylene insertion up to 3 months	–	Good biocompatibility	[142]
Enzymatic G-BFC	0.52 V	1.9 μW/mL	1 week	pH:7.2 Glucose: 0–15 mM	Inconclusive	[143]
Nanozymatic G-BFC	–	44 mW/cm^2^	20 cycles of charging-discharging	pH: 7 Glucose: 0–0.1 M	Inconclusive	[144]

## 3 Conclusion and future prospects

Among biofuel cells, microbial fuel cells have been extensively studied and used in various applications, and their power output remains relatively modest compared to enzymatic biofuel cells. Enzymatic biofuel cells, especially nanozymatic variants, show potential for higher power outputs due to advancements in nanotechnology and enzyme engineering.

Among the various enzymatic biofuel cells, the field of glucose biofuel cells (G-BFCs) shows the most promise for use as future surgical implants (cardiac pacemakers, biosensors, etc.), provided challenges related to extended performance and stability can be overcome related to their more extended performance over time and their stability. G-BFCs' performance primarily depends on the electron transfer mechanism in their applied environment. The mediator electron transfer (MET) process provides higher power output and current density but at the expense of larger device sizes compared to those relying on direct electron transfer (DET). The mediators' surface nature is crucial for improving the performance of enzymatic reactions. Organometallic mediators may undergo loss of ligands, whereas, in contrast, organic mediators may undergo dimerization, thus contributing to instability in the power output of a G-BFC, potentially causing hazardous substances that cause inflammation in the human body.

The performance of G-BFCs is influenced by several factors: enzyme efficiency, which depends on the choice and stability of enzymes such as glucose oxidase; the materials used for electrodes, with nanostructures and catalysts like platinum enhancing electron transfer; and the characteristics of membranes that need to allow glucose while blocking other substances selectively. Environmental conditions such as pH and temperature also play a crucial role. Different regulation strategies are employed to enhance performance. These include enzyme immobilization techniques to improve stability, the use of nanotechnology to increase surface area and catalytic properties, optimized electrode designs, genetic engineering of enzymes for better performance, and hybrid systems combining enzymatic and non-enzymatic catalysts. However, several challenges must be addressed for G-BFCs to be viable in cardiac pacemakers. These include achieving sufficient power density, ensuring long-term stability in the physiological environment, maintaining biocompatibility to prevent adverse reactions, miniaturizing the cells to fit within the limited space of pacemakers, and ensuring stable performance despite physiological fluctuations.

Solutions to these challenges involve advanced enzyme engineering to develop more robust enzymes, novel electrode materials like graphene and carbon nanotubes to enhance conductivity and surface area, encapsulation techniques to protect sensitive components, integrating G-BFCs with other power sources for reliable energy supply, and smart regulation systems that adjust the biofuel cell's operation based on real-time physiological conditions. Research advances in these areas aim to make G-BFCs a practical and reliable power source for medical implants.

The integration of glucose-based biofuel cells in medical devices for cardiac activities represents a significant advancement in medical technology. By providing a sustainable and biocompatible power source, G-BFCs can potentially improve management of heart diseases. Continued research, strategic partnerships, and focused commercialization efforts are essential to realize this potential and bring these innovative solutions to the market.

Several factors, such as technological readiness, advances in material science, and regulatory approval, including successful clinical trials and certifications for market entry, drive the commercialization of glucose-based biofuel cells in medical devices. Ongoing research is focused on enhancing their performance and scalability. Collaboration between biotechnology firms, medical device manufacturers, and research institutions accelerates the development and commercialization process. Venture capital and healthcare fund investments are pivotal in scaling production and marketing efforts. The increasing prevalence of heart diseases globally creates a substantial market for innovative medical devices. G-BFCs-powered devices offer unique selling points such as reduced maintenance, longer operational life, and improved patient outcomes, driving market demand. Efforts to enhance the power density and stability of G-BFCs, address biofouling, and ensure long term biocompatability are ongoing and crucial. The cost of production and integration of G-BFCs must be competitive with existing battery technologies. Economies of scale and technological advancements are essential to reducing costs.

From the review of the published works, it is concluded that the promising potential ofG-BFCs' to power medical implants relies on developing effective enzyme-to-electrode electron transfer processes and reducing the size of G-BFCs. Recent advancements in circuit arrangements have also shown improved power output, allowing for more convenient implant designs.

## Data availability

No datasets were generated or analyzed during the current study.

## CRediT authorship contribution statement

**Indrani Chakraborty:** Writing – original draft. **Richard T. Olsson:** Writing – original draft, Writing – review & editing. **Richard L. Andersson:** Writing – review & editing. **Annu Pandey:** Conceptualization, Writing – original draft.

## Declaration of competing interest

None.
